# Depression and anxiety disorder among epileptic people at Amanuel Specialized Mental Hospital, Addis Ababa, Ethiopia

**DOI:** 10.1186/s12888-015-0589-4

**Published:** 2015-09-02

**Authors:** Minale Tareke Tegegne, Tilahun Belete Mossie, Andargie Abate Awoke, Ashagre Molla Assaye, Belete Temitm Gebrie, Desalegn Asmare Eshetu

**Affiliations:** 1 College of Medicine and Health Science, Bahir Dar University, Bahir Dar, Ethiopia; 2 College of Health Science, Nursing Department, Psychiatry Unit, Mekelle University, Mekelle, Ethiopia; 3 College of Public Health and Medical Sciences, Department of Nursing, Jimma University, Jimma, Ethiopia; 4 Amhara National Regional State Health Bureau, Bahir Dar, Ethiopia; 5 Debre-Birhan Referral Hospital, Debre-Birhan, Ethiopia

## Abstract

**Background:**

Although depression and anxiety disorders are very common in people with epilepsy; there are no studies that assessed the magnitude and associated factors among epileptic people in Ethiopia. Therefore, this study determined prevalence and associated factors of depression and anxiety disorders in people with epilepsy.

**Method:**

An institution based cross-sectional study was conducted from April to May, 2013, among 423 people with epilepsy from the outpatient department of Amanuel Mental Specialized Hospital. Depression and anxiety were assessed using the Hospital Anxiety and Depression Scale. Logistic regression analysis was used to assess predictors of depression and anxiety.

**Results:**

The prevalence of anxiety and depression among epileptic people were 33.5 and 32.8 %, respectively. Monthly income, frequency of seizure and side effects of anti convulsants were found to be significantly associated with both depression and anxiety. Being divorced/widowed was associated with anxiety while using poly-therapy of anti convulsants, perceived stigma, and inability to read or write were associated with depression.

**Conclusion:**

The prevalence of co-morbid anxiety and depression was found to be high among people with epilepsy. Early identification of co-morbid depression and anxiety in people with epilepsy and managing epilepsy to become seizure free should be of great concern for health care providers.

## Background

Epilepsy is a common neurological condition characterized by recurrent and unpredictable seizures associated with significant psychological and social consequences. More than eighty percent of 50 million people living with epilepsy (PWE) worldwide reside in low-income regions where human and technological resources for health care are extremely limited. Although epilepsy is more likely to be diagnosed in childhood or elder years, it is not confined to any age group, sex or race. Frequent seizures may cause difficulties in important areas of life (at school or the workplace), as well as sometimes hindering the development of new friendships and relationships. Fortunately, epilepsy can be managed with antiepileptic medications and more than 70 percent of people become seizure-free with treatment [1–3].

Depression is characterized by loss of interest, depressed mood, disturbance of sleep, problem in appetite and psychomotor activity, difficulty to concentrate or make decision, guilty or sinful feeling, easily tiredness and recurring thoughts of death or suicide [4]. Anxiety is the presence of fear or apprehension that is out of proportion to the context of the life situation. An anxiety disorder can be expressed in different ways such as uncontrollable worry, intense fear (phobias or panic attacks) or upsetting dreams or flashbacks of a traumatic event [4, 5].

Depression and anxiety disorders are very common in people with epilepsy. The altered brain activity that causes epileptic seizures can lead to depressive moods and the stress of living with a chronic condition can worsen feelings of depression and anxiety. As a consequence, epilepsy may be more difficult to manage as depression is sometimes known to make seizures more frequent and can decrease the motivation to manage epilepsy effectively [6].

People with epilepsy experience depression at two to three times the rate of the general population [7]. Literatures reported a strong link between epilepsy, depression and anxiety disorders. The disorders can be aggravated or developed following being diagnosed for epilepsy; or as a consequence of living with epilepsy. Structural brain abnormalities, monoamine pathways, cerebral glucose metabolism and the hypothalamic-pituitary-adrenal axis are also associated with the pathogenesis of depression in people with epilepsy. But once they are diagnosed, depression and anxiety disorders can be safely and effectively treated at the same time as epilepsy. Treatments for mental health conditions can greatly improve quality of life and can reduce the frequency and impact of seizures [8, 9].

According to different world wide literatures the magnitude of depression and anxiety disorder among adult epileptic people had a varied figure. The presence of depressive symptoms accounted 20 % in Thailand [10], 25 % in India [11], 24 to 32 % in Brazil [12, 13], 38 and 60 % at China and Pakistan, respectively [14, 15]; where as it was 25.5 % in Egypt [16]. In addition, anxiety disorders were reported among 39 % of Thailand subjects [10], 33 to 39 % of Brazilian participants [12, 13], 30 % of Chinese [14] and 47 % of Egyptians [16].

There are multiple risk factors associated with increased risk of psychiatric problems in epilepsy which can be broadly divided into biological (e.g. type and severity of epilepsy), psychosocial and iatrogenic (antiepileptic drugs, surgery) [17].

On the other hand, different factors were reported as significant factors for co-existence of depression and anxiety disorder among epileptic people. Female gender, frequent seizures, perceived stigma, low schooling, suicidal thought, or attempt, poly therapy and poor seizure control were risk factors for anxiety disorders [10, 12, 13, 16]. Similarly, having frequent seizure, head trauma, perceived stigma, lower educational level, suicidality [12, 13], male gender, being married, low socio economic status, uncontrolled seizure and poly therapy were significantly associated with depression [15].

There are limited studies which showed the magnitude of depression and anxiety among epileptic patients in Sub-Saharan region particularly in Ethiopia. The aim of this study was to determine the magnitude of depression and anxiety disorders among epileptic people at Amanuel Mental Specialized Hospital, Ethiopia. Hence, the findings might have importance to stakeholders and policy makers working in Neuro-Psychiatric areas by showing its prevalence and the factors associated with it.

## Methods

### Study settings and population

A cross sectional study design was conducted at Amanuel Mental Specialized Hospital (AMSH) in Addis Ababa from April to May, 2013. AMSH is one of oldest hospitals established in 1937 and located in western part of Addis Ababa, the capital city of Ethiopia. Ethiopia is the second-most populous country in Sub-Saharan Africa with a population of more than 85 million people. It is an agrarian country and agriculture accounts for 43 percent of the gross domestic product. Primary health service coverage has reached 93 % with 127 hospitals, 3,245 health centers, 16,048 health posts and more than 4,000 private for profit and not for profit clinics [18]. In Ethiopia, epilepsy is often mistakenly seen as a form of mental illness by the community and is usually treated by psychiatrists and psychiatric nurses. The most prescribed antiepileptic drugs were phenobarbitone, phenytoin and sometimes carbamazepine and sodium valproate. The hospital gives service for approximately 1200 epileptic patients per month.

The Study population was epileptic patients who had treatment follow up at outpatient department during study period. Patients aged 18 years and above with diagnosis of epilepsy and under treatment with one or more antiepileptic drugs from the outpatient units for at least 6 months were included in the study. Patients with serious general medical condition and unable to communicate were excluded.

### Sample size and sampling procedures

The sample size for the study was calculated using the formula [*n* = ((zα/2)2 p (1-p))/ d2] for estimating a single population proportion at 95 % confidence interval (CI) (Zα/2 = 1.96), 5 % margin of error. Due to absence of data in the country; proportion of population living with epilepsy and who had depression and anxiety was assumed to be 50 %, and by adding 10 % contingency for non response rate, a total of 423 study populations were involved.

Systematic sampling method was used to select study population coming to AMSH during the study period from 1200 epileptic people. AMSH has 13 outpatient departments from which two of them are neurologic clinics. Sampling interval was determined by dividing total study population who had follow up during one month data collection period (1200) by total sample size (423).The sampling fraction is: 1200/423 ≈ 3. The first individual was selected by lottery method, and the next respondent was chosen at regular intervals (every 3^rd^) by data collectors.

### Data collection and quality control

The data was collected using a pretested structured questionnaire developed in English and translated to Amharic and then to English by expertise and senior psychiatrist to ensure its consistency. The questionnaire was pre-tested on 5 % of the sample size at AMSH two days prior to data collection. The questionnaire included socio-demographic characteristics and clinical factors of epilepsy. Concerning co-morbid anxiety, depression and stigma, Hospital Anxiety and Depression Scale (HADS) and stigma scale were used, respectively. HADS is a 14-item questionnaire, commonly used to screen for symptoms of anxiety and depression. The 14-items can be separated into two 7-item sub-scales for anxiety and depression. HADS scale was validated in Ethiopia and the internal consistency was 0.78 for the anxiety, 0.76 for depression subscales and 0.87 for the full scale. The scales use a cut -off score for anxiety and depression of greater than or equal to 8 [19]. Felt stigma was assessed by three-item stigma scale which is validated at Zambia and comprised of dichotomous questions in which a positive response is indicative of felt or perceived stigma with an overall possible score ranging from 0 (no felt stigma) to 3 (maximally felt stigma) [20]. Data was collected by three trained diploma psychiatry nurses and one supervisor (BSc nurse) for a period of one month. Face to face interview was employed using local Amharic language.

Two days training was given to orient data collectors and supervisor on the questionnaire to be used, the purpose of the study and how to approach respondents and obtain consent. The data collectors were supervised daily and the filled questionnaires were checked for completeness and consistency by supervisor and principal investigator.

### Data management and analysis

The data was coded, checked, cleaned and entered into computers using software Epi Info version 3.5.4 and then exported into SPSS window version 20 for analysis. Frequency, percentage, median were used to describe relevant variables using tables and graphs. Logistic regression was performed to assess the association between binary outcomes and different explanatory variables. Bivariate analysis was first conducted for each potentially explanatory risk factor. Variables that satisfied *p*-value < 0.2 were selected for further analysis using multivariate logistic regression analysis in order to control confounding effects.

The strength of association was interpreted using odds ratio (OR) and confidence interval (CI). *P*-value < 0.05 was considered statistically significant in this study.

### Ethical consideration

Ethical clearance was obtained from University of Gondar and AMSH. Informed consent was obtained from the respondents. They were given the right to refuse to take part in the study as well as to withdraw at any time during the interview process. Confidentiality was maintained throughout the study.

## Results

### Socio-demographic characteristics of the respondents

Of the total 423 study participants enrolled, 415 were interviewed yielding a response rate of 98 %. Respondents’ age ranged from 18 to 72 years with a median age of 28 years. Around fifty five percent of the respondents were males. Of the total respondents, 42.7 % were in the age group of 25–34 years. Majority of the respondents were single (61.4 %), urban dwellers (84.6 %), orthodox followers (67.0 %), having job (78.1 %), and Amhara (38.1 %) followed by Oromo (29.2 %) by ethnicity. Regarding educational status, 37.6 % attended secondary school followed by primary school (36.4 %) (Table 1).

**Table 1:** Distribution of respondents by socio-demographic characteristics at AMSH, Addis Ababa, 2013 (*n* = 415)

Variable		Number	Percentage (%)
Sex	Male	229	55.2
	Female	186	44.8
Age	18-24	130	31.3
	25-34	177	42.7
	35-44	68	16.4
	45+	40	9.6
Marital status	Single	255	61.4
	Married	146	35.2
	Divorced/Widowed	14	3.4
Residence	Urban	351	84.6
	Rural	64	15.4
Religion	Orthodox	278	67.0
	Muslim	84	20.2
	Protestant	47	11.3
	All others	6	1.4
Ethnicity	Amhara	158	38.1
	Oromo	121	29.2
	Gurage	93	22.4
	Tigre	26	6.3
	Others	17	4.1
Educational status	Unable to write and read	35	8.4
	Primary school	151	36.4
	Secondary school	156	37.6
	Diploma and above	73	17.6
Occupation	With Job	324	78.1
	Without Job	91	21.9
Monthly income (ETB)	<200	117	28.2
	200-500	110	26.5
	501-1000	103	24.8
	>1000	85	20.5

### Clinical characteristics of the respondents

Out of the total 415 respondents, 35.2 % were with epilepsy for five years followed by eleven years and above (34.2 %). Concerning age at onset of illness, 38.8 % found between 10–19 years and 20–29 years (35.4 %). Most of the respondents (54.9 %) had one or more seizure attacks per month, under monotherapy (single antiepileptic drug) (61.9 %) and had no side effects of antiepileptic drugs (52.5 %). Around forty percent of the respondents perceived that they were stigmatized by other people because of their illness (Table 2).

**Table 2:** Distribution of respondents by clinical factors at AMSH, Addis Ababa, 2013 (*n* = 415)

Variable name		Frequency	Percent
Duration of illness	≤5 years	146	35.2
	6-10years	127	30.6
	≥11 years	142	34.2
Age at onset of illness	<10 years	53	12.8
	10-19 years	161	38.8
	20-29 years	147	35.4
	≥30 years	54	13.0
Frequency of seizure	≥1/month	229	54.9
	1-3/year	58	14.0
	Seizure free for 1 year	129	31.1
Type of drugs	One	257	61.9
	≥two	158	38.1
Side effects of Anti-epileptic drugs (AEDs)	Yes	197	47.5
	No	218	52.5
Medication duration	≤5 years	232	55.9
	6-10 years	79	19.0
	≥11 years	104	25.1
Perceived stigma	Yes	169	40.7
	No	246	59.3
Ever use substance	Yes	53	12.8
	No	362	87.2
current use substance	Yes	29	7.0
	No	386	93.0

### Prevalence of depression and anxiety among epileptic patients

**Fig. 1:**
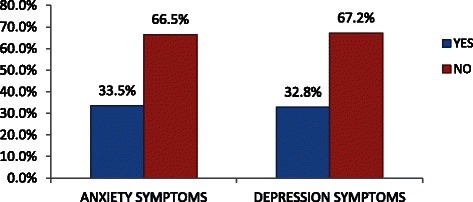
Frequency of depression and anxiety symptoms among epileptic people attending at AMSH, 2013

The prevalence of anxiety symptoms among epileptic patient was found to be 33.5 % whereas prevalence of depression was 32.8 % (Fig. 1).

### Factors associated with co-morbid anxiety and depression

On bivariate analysis, the factors found to fulfill the minimum requirement (*p*-value < 0.2 in this study) were age, marital status, educational level, occupation, monthly income, frequency of seizure, types of drugs, side effects of antiepileptic drugs (AEDs) and perceived stigma. However, no significant association was noted in depression and anxiety between males and females among epilepsy patients. Moreover, no differences were found among study variables like residence, duration of illness, both ever and current use of psychoactive substance at minimum requirement.

The results of multivariate logistic regression showed that monthly income, frequency of seizure and side effects of AEDs were significantly associated with both depression and anxiety. However, being divorced/widowed was significantly associated with anxiety where as perceived stigma, types of AEDs, and unable to read and write were associated with depression.

It was found that lower educational level was significantly associated with depression. Being unable to read and write was four times (AOR = 4.36, 95 % CI: 1.33, 14.21) more likely to have depression as compared to respondents with educational level of diploma and above.

The result indicated that patients who had low income were about four and three times more likely to have depression and anxiety, respectively as compared to patients who had high income (AOR = 4.14, 95 % CI: 1.67, 10.25 and AOR = 3.01, 95 % CI: 1.52, 5.96).

Frequency of seizure was another factor associated with both depression and anxiety. Those who had seizure frequency greater than or equal to one seizure/month were three times and twice more likely to have depression and anxiety as compared to patients with no seizure in one year period, respectively (AOR = 3.55, 95 % CI: 1.82, 6.93 and AOR = 2.21, 95 % CI: 1.28, 3.79).

Moreover, patients who perceived stigma were twice more likely to have depression as compared to patients who didn’t perceived stigma (AOR = 1.77, 95 % CI: 1.07, 2.93). Similarly, patients who were taking two or more types of AEDs were twice more likely to have depression as compared to patients who were taking one type of AEDs (AOR = 1.85, 95 % CI: 1.08, 3.17). Regarding side effects of medication, those who had side effects of AEDs were around three times more likely to have co-morbid anxiety and depression (AOR = 2.51, 95 % CI: 1.60,3.94) and AOR = 3.07, 95 % CI: 1.80,5.21, respectively) (Tables 3 and 4).

**Table 3:** Bivariate and multivariate analysis of variables associated with anxiety among epileptic patients at AMSH, Addis Ababa Ethiopia, 2013 (*n* = 415)

Variable		Anxiety			
		Yes	No	COR(95 % CI)	AOR(95 % CI)
Marital status	Married	41	105	1	1
	Single	88	167	1.39(0.86,2.10)	1.22(0.75,1.96)
	Divorced/widowed	10	4	6.40(1.90,21.56)	6.27(1.69,23.16)^**^
Education	Unable to read &write	19	16	2.58(1.12,5.91)^*^	1.08(0.38,3.06)
	primary school	47	104	0.98(0.53,1.79)^*^	0.54(0.25,1.16)
	Secondary school	50	106	1.02(0.56,1.86)	0.65(0.32,1.33)
	Diploma &above	23	50	1	1
Occupation	With Job	97	227	1	1
	Without Job	42	49	2.00(1.24,3.22)^*^	1.14(0.61,2.14)
Monthly income	<200	55	62	3.82(1.98,7.35)^*^	3.01(1.52,5.96)^**^
	200-500	42	68	2.66(1.36,5.18)^*^	2.24(1.11,4.49)^**^
	501-1000	26	77	1.45(0.72,2.93)^*^	1.44(0.68,3.04)
	>1000	16	69	1	1
Frequency of attack	≥1/month	96	132	2.88(1.74,4.77)^*^	2.21(1.28,3.79)^**^
	1-3/year	17	41	1.64(0.80,3.34)^*^	1.41(0.67,2.99)
	Seizure free/year	26	103	1	1
Type of drugs	One	66	191	1	1
	≥ two	73	85	2.48(1.63,3.78)^*^	1.49(0.91,2.46)
Side effects of AED	Yes	92	105	3.18(2.07,4.88)^*^	2.51(1.60,3.94)^**^
	No	47	171	1	1
Perceived stigma	Yes	75	94	2.26(1.49,3.44)^*^	1.38(0.86,2.20)
	No	64	182	1	1

Note: *P < 0.2 **P < 0.05

**Table 4:** Bivariate and Multivariate analysis of variables associated with depression among epileptic people at AMSH, Addis Ababa Ethiopia, 2013 (*n* = 415)

Variable		Depression			
		Yes	No	COR(95 % CI)	AOR(95 % CI)
Age	18-24	30	100	0.79(0.35,1.76)	0.75(0.25,2.17)
	25-34	66	111	1.56(0.73,3.34)	2.29(0.83,6.31)
	35-44	29	39	1.96(0.84,4.56)	2.29(0.76,6.87)
	45+	11	29	1	1
Education	Unable to read &write	21	14	6.92(2.80,17.09)^*^	4.36(1.33,14.21)^**^
	primary school	48	103	2.15(1.07,4.29)^*^	1.44(0.61,3.40)
	Secondary school	54	102	2.44(1.23,4.84)	2.52(0.90,5.81)
	Diploma &above	13	60	1	1
Occupation	With Job	93	231	1	1
	Without Job	43	48	2.22(1.38,3.58)^*^	1.25(0.62,2.54)
Monthly income	<200	52	65	5.38(2.59,11.17)^*^	4.14(1.67,10.25)^**^
	200-500	44	66	4.48(2.14,9.39)^*^	4.17(1.69,10.24)^**^
	501-1000	29	74	2.63(1.22,5.66)^*^	3.34(1.37,8.10)^**^
	>1000	11	74	1	1
Frequency of attack	≥1/month	104	124	5.92(3.30,10.63)^*^	3.55(1.82,6.93)^**^
	1-3/year	16	42	2.69(1.23,5.85)^*^	1.83(0.76,4.41)
	Seizure free/year	16	113	1	1
Type of drugs	One	57	200	1	1
	≥ two	79	79	3.50(2.28,5.38)^*^	1.85(1.08,3.17)^**^
Side effects of AED	Yes	97	100	4.45(2.85,6.94)^*^	3.07(1.80,5.21)^**^
	No	39	179	1	1
Perceived stigma	Yes	78	91	2.77(1.82,4.23)^*^	1.77(1.07,2.93)^**^
	No	58	188	1	1

Note: *P < 0.2 **P < 0.05

## Discussion

In this study, the prevalence of anxiety among epileptic people was 33.5 % and the prevalence of depression was 32.8 %. The prevalence of anxiety was in line with the study done in China (30 %) and Brazil (33-39 %) [12, 14]. However, in the present study, it was lower than the result reported from Thailand (39 %) and Egypt (47 %) [10, 16]. The possible reason for the difference might be the inclusion criteria in Thailand were age between 15–50 years and could read and fill with self-administered questionnaire, while in Egypt there was instrument difference which is beck depression inventory and HAM-A and exclude any substance use, neurologic disease other than epilepsy.

The prevalence of depression (32.8 %) among epileptic people, in this study, is higher than the result (20 %) reported from Thailand [10] and lower than the result (60 %) in Pakistan [15]. Methodological issues might have contribution for differences found, for example, in Thailand; only those who could read and communicate were included. Education is one of the associated factors of depression as indicated in our study which is also in line with study done in Jimma [19]. Furthermore, the difference in sampling procedures and diagnostic instrument for depression and anxiety (different scales) might have a role for the variation.

Different studies indicated that people with low economic status and participants in low educational level had a higher risk of depression and anxiety [21–23]. The results of these studies are consistent with our study.

Regarding clinical factors, side effects of AEDs and frequency of seizure were associated with co-morbid depression and anxiety in this study. People who had side effects of medication and frequent seizure were found to have depression and anxiety as compared to people who had no side effects of medication and no seizure. This finding was consistent with many previous studies [9, 24].

The results of the present study also revealed that depression is significantly influenced by felt stigmatization about their epilepsy; which is in line with study done in Mangalore city of south India [25].

The strength of this study is the first of its kind in Ethiopia that determined the prevalence and associated factors for both depression and anxiety among epileptic patients. However, our limitations include recall bias regarding duration of illness, age at the onset of seizure and medication duration. Some important variables such as intellectual disability and treatment adherence were not assessed.

## Conclusion

The prevalence of co-morbid anxiety and depression was found to be high among epileptic people in this study. Unable to read and write, low monthly income, frequent attacks of seizure, treatment with two or more drugs, side effects of AEDs and perceived stigma were significantly associated with co-morbid anxiety and depression. Clinicians and neurologists should early identify co-morbid psychiatric illnesses like depression and anxiety in people with epilepsy and it should be of great concern for health care providers. Further research should be done in different part of the country to provide stronger evidence regarding the prevalence and factors associated with this co-morbid anxiety and depression among epileptic people.
